# Correlation between OSAHS and Early Peripheral Atherosclerosis Indices in Patients with Type 2 Diabetes Mellitus in China: A Cross-Sectional Inpatient Study

**DOI:** 10.1155/2021/6630020

**Published:** 2021-02-10

**Authors:** Xin Zhao, Xiaofeng Yu, Sixu Xin, Wei Zhang, Xiaomei Zhang, Linong Ji

**Affiliations:** ^1^Endocrinology Department, Peking University International Hospital, Beijing, China; ^2^Sleep Center Department, Peking University International Hospital, Beijing, China; ^3^Endocrinology Department, cc, Beijing, China

## Abstract

Objective: To analyze the differences of early atherosclerosis indices in type 2 diabetes mellitus (T2DM) patients with different degrees of obstructive sleep apnea-hypopnea syndrome (OSAHS) and explore the correlation between them, so as to provide a new clinical basis for the prevention and treatment of early atherosclerosis in patients with T2DM and OSAHS. *Methods*. A prospective study was conducted in 312 patients with T2DM and snoring who were hospitalized in the Department of Endocrinology, Peking University International Hospital from January 2017 to January 2020. According to the monitoring results, 312 patients were divided into 4 groups including the control group (208 cases), mild OSAHS group (18 cases), moderate OSAHS group (38 cases), and severe OSAHS group (48 cases). Multivariate logistic regression analysis was used to analyze the early atherosclerosis indices including brachial-ankle pulse wave velocity (PWV) and ankle-brachial index (ABI) in patients with T2DM coexistence with different degrees of OSAHS. *Results*. (1) As the degree of OSAHS increased, ABI decreased gradually and was lower than that in the control group, but PWV increased and was higher than that in the control group (*p* < 0.05, respectively). (2) The apnea-hypopnea index (AHI) positively correlated with PWV (r = 0.36, *p* < 0.05) and negatively correlated with ABI (*r* = −0.37, *p* < 0.05). (3) Multivariate logistic regression showed that after adjusting for age, gender, duration, BMI, blood pressure, blood glucose, blood lipid, and other factors, OSAHS was a risk factor of lower extremity arterial disease (LEAD) in patients with T2DM. With the increase of degree of OSAHS, the risk of lower extremity atherosclerosis gradually increased. *Conclusion*. OSAHS is an independent risk factor of LEAD in patients with T2DM, and with the increase of AHI, the ABI and PWV have changed, which provides a new clinical basis for the prevention and the treatment of early atherosclerosis in patients with T2DM and OSAHS.

## 1. Introduction

The prevalence of type 2 diabetes mellitus (T2DM) in China is increasing year by year. The main reasons of death in patients with T2DM are complications such as lower extremity arterial disease (LEAD) [1]. Obstructive sleep apnea-hypopnea syndrome (OSAHS), as a common disease of sleep disordered breathing, has gradually attracted attention in recent years. OSAHS and T2DM often coexist. The incidence rate of OSAHS in T2DM patients is 18-36%, while in OSAHS patients, the incidence rate of T2DM is about 40% [2].

Atherosclerosis is a systemic disease involving the thickening and hardening of the arterial walls. Patients with T2DM are more likely to develop atherosclerotic diseases, and nearly 75% of deaths in patients with T2DM is directly due to atherosclerotic diseases, with coronary heart disease posing the highest mortality [3].In recent years, it has been reported that the prevalence of diabetic macrovascular and microvascular complications in T2DM patients with OSAHS is significantly increased [4, 5]. However, a limited number of studies have reported on whether there is a correlation between the early peripheral atherosclerosis indices and OSAHS in patients with T2DM. The indices including the ankle-brachial index (ABI) and brachial-ankle pulse wave velocity (PWV) are noninvasive indicators for assessing the early changes of atherosclerosis.

The purpose of this study is to analyze the differences and changes of early atherosclerosis indices in T2DM patients with different degrees of OSAHS and aimed at providing a new clinical basis for the prevention and treatment of atherosclerosis in these patients.

## 2. Materials and Methods

### 2.1. Patients and Study Design

This is a prospective study. 312 patients including 208 males and 104 females with T2DM and snoring who were hospitalized in the Department of Endocrinology, Peking University International Hospital from January 2017 to January 2020 were enrolled in this study. The T2DM diagnostic criteria were based on the 1999 World Health Organization's diagnostic criteria [6], including (1) random blood glucose levels ≥ 11.1 mmol/l, (2) fasting blood glucose levels ≥ 7.0 mmol/l, and (3) OGTT test with blood glucose levels ≥ 11.1 mmol/l at 2 hours after receiving 75 g of glucose. In the absence of diabetic symptoms, the OGTT test was repeated on the patient the next day to confirm. If one or more of the three criteria were met, the patient was diagnosed with DM. In addition, according to the clinical classification, the patient was diagnosed with T2DM. The exclusion criteria for this study included a history of end-stage renal disease, cancer, stroke, cardiovascular disease, or hormone-related endocrine disease, lower extremity vascular occlusions, or arterial calcification disease. This study was approved by Bioethics Committee of Peking University International Hospital. All participants have signed the informed consent form.

### 2.2. Polysomnography (PSG) Examination

None of the subjects had been diagnosed with OSAHS, and all the patients carried out the all-night PSG monitoring in the sleep monitoring center of Peking University International Hospital. The monitoring indices included the electroencephalogram (EEG) which was obtained with C4A1, C3A2, 01A2, and 02A1 leads; electrooculogram (EOG); mandibular mental electromyography (EMG); electrocardiogram (ECG); respiratory airflow; thoracoabdominal respiratory movement; blood oxygen saturation (SaO_2_); body position; snoring; and EMG of tibial anterior muscle. Sleep monitoring time was not less than 7 hours at night. Patients were asked not to use sedatives, coffee, wine, or strong tea on the day of sleep monitoring. The apnea-hypopnea index (AHI) means the sum of the average number of apnea and hypopnea per hour.

According to the Chinese diagnostic criteria of OSHAS [7], Apnea and hypopnea recurred more than 30 times or AHI ≥ 5/h during 7 hour-sleep. Apnea events were mainly obstructive, accompanied by snoring, sleep apnea, daytime sleepiness, and other symptoms.

According to AHI, patients were divided into four groups: the control group, mild group (AHI was 5-15/h), moderate group (AHI was 16-30/h), and severe group (AHI > 30/h).

### 2.3. Medical Records and Clinical Data

Medical history and relevant clinical indices were recorded, including age, gender, height, weight, systolic blood pressure (SBP), diastolic blood pressure (DBP), and diabetes duration. From the medical records, the body mass index (BMI) was calculated by the formula weight/height^2^ (kg/m^2^).

### 2.4. Laboratory Tests

The patients were required to fast for 8 hours prior to blood collection. The blood tests included fasting blood glucose (FBG), glycosylated hemoglobin (HbA1c), serum creatinine (sCr), uric acid (UA), total cholesterol (TC), triglycerides (TG), low-density lipoprotein cholesterol (LDL-C), and high-density lipoprotein cholesterol (HDL-C). Postprandial blood glucose (PBG) was collected 2 h after a mixed nutrient load. All blood tests were performed at Peking University International Hospital. FBG, PBG, sCr, UA, TC, TG, LDL-C, and HDL-C were measured by using enzyme-linked immunosorbent assay methods, while HbA1c was measured by high-performance liquid chromatography (HPLC). Glomerular filtration rates (eGFR) were calculated using the sCr levels according to the CKD-EPI-ASIA equation as follows:

For males,

(1) $$\begin{array}{c} sCr\leq 0.9mg/dl:{eGFR}_{CKD-EPI-ASIA}=141\times \left(sCr/0.9\right)-0.411\times 0.993^{age}\times 1.057,\\ sCr>0.9mg/dl:{eGFR}_{CKD-EPI-ASIA}=141\times \left(sCr/0.9\right)-1.209\times 0.993^{age}\times 1.057. \end{array}$$

For females,

(2) $$\begin{array}{c} sCr\leq 0.7mg/dl:{eGFR}_{CKD-EPI-ASIA}=141\times \left(sCr/0.7\right)-0.329\times 0.993^{age}\times 1.049,\\ sCr>0.7mg/dl:{eGFR}_{CKD-EPI-ASIA}=141\times \left(sCr/0.7\right)-1.209\times 0.993^{age}\times 1.049. \end{array}$$

### 2.5. Early Atherosclerotic Indices

ABI (ankle-brachial index) and PWV (brachial-ankle pulse wave velocity) measurements were used to assess the degree of atherosclerosis. The BP-203 III automatic atherosclerosis analyzer (Omron, Tokyo, Japan) was used to measure the ABI and PWV values. Patients were allowed to rest for 5 min before measuring the systolic pressure of the right and left anterior tibial artery (ankle) and the right and left brachial artery (brachial artery). The left and right ABI were calculated as the systolic ankle pressure divided by the systolic brachial pressure. PWV was calculated as the time interval between the anterior tibial artery (ankle) and the initial segment of the pressure wave for the brachial artery and the distance between the two selected components. ABI and PWV values were measured and assessed by the same group of physicians in the Department of Endocrinology to avoid potential interexaminer differences. The ABI and PWV averages were calculated by determining the mean value between the left and right ABI and PWV.

### 2.6. Doppler Ultrasonography for Measuring LEAD

The arteries of both lower extremities were examined by ultrasound professional technician using lower extremity arterial color Doppler ultrasonography (Phillips iE33, Washington, DC, USA). The patients were in supine position, and bilateral lower extremity arteries (total femoral, femoral deep, superficial, popliteal, anterior tibial, posterior tibial, dorsum of foot) were examined. The examination contents included artery diameter and intimamedia thickness of lower extremity artery, whether there was plaques and whether there was vascular stenosis. The coefficient of variance was 1.92%. According to the ultrasound results, if the patient has lower extremity artery stenosis or occlusion, the patient is diagnosed as LEAD.

### 2.7. Statistical Analysis

Statistical analysis was performed with the SPSS Version 21.0 software (IBM, Chicago, IL, USA). The data were analyzed using the Kolmogorov-Smirnov test, and all variables had a normal distribution and were expressed as the mean ± standard deviation. Multigroup comparisons of the sample were compared with the one-way analysis of variance (ANOVA). The least significant difference (LSD) method was used to compare the statistical significance between the groups. Count data were compared as ratios statistically, and the *χ*^2^ test was used for comparison among the four groups. Pearson correlation analysis and multivariate linear regression were used to assess the association between ABI, PWV, and AHI. Multivariate logistic regression was used to assess the factor of LEAD in T2DM patients with OSAHS. *p* values <0.05 were considered statistically significant.

## 3. Results

### 3.1. Comparison of General Characteristics, Biochemical Indices, PSG Indices, and Atherosclerotic Indices among the Four Groups

Compared with the control group, the patients with OSAHS were older and had higher BMI. There were significant differences among the four groups (*p* < 0.05, respectively). Among the four groups, SBP of patients with OSAHS was significantly higher than that of the control group, and the UA level was significantly higher than that of the control group (*p* < 0.05, respectively). At the same time, the results showed that ABI in the OSAHS group was lower than that in the control group, while PWV was higher in the OSAHS group than that in the control group (*p* < 0.05, respectively). The proportion of OSAHS coexisted with LEAD was higher, and as the severity increasing of OSAHS, the proportion with LEAD increased gradually (*p* < 0.05). There was no significant difference in the gender ratio, diabetic duration, blood lipid, blood glucose, UACR, and eGFR among the four groups (*p* > 0.05, respectively) (shown as Table 1).

### 3.2. Correlation Analysis between AHI Level and General Characteristics, Biochemical Indices, ABI, and PWV

Age, BMI, TC, TG, LDL-C, ABI, and PWV levels were positively significantly correlated with AHI (*p* < 0.05, respectively). ABI was negatively correlated with AHI (*r* = −0.37, *p* < 0.05), while PWV positively correlated with AHI (*r* = 0.36, *p* < 0.05) (shown as Table 2).

### 3.3. Multiple Stepwise Linear Regression Results among AHI, ABI, and PWV

With ABI and PWV as dependent variables, respectively, AHI, age, gender, BMI, diabetic duration, blood glucose, blood pressure, blood lipid, UA and other indices as independent variables, and multiple linear regression model were established. The results showed that after adjusting for age, gender, BMI, diabetic duration, blood glucose, blood pressure, blood lipid, and UA, AHI was an independent risk factor for decreased ABI and increased PWV (shown as Table 3).

### 3.4. Multivariate Logistic Regression Results of LEAD in T2DM Patients with OSAHS

With LEAD as a dependent variable, the results showed that AHI was an independent risk factor for LEAD in patients with T2DM.After adjusting for age, gender, BMI, diabetic duration, blood glucose, blood pressure, blood lipid, UA, and other indices, AHI was still an independent risk factor for LEAD in patients with T2DM. With the increasing degree of OSAHS, the risk of LEAD in patients increased gradually (shown as Table 4).

Model 2 is adjusted for age, gender, diabetic duration, BMI, blood glucose, blood pressure, blood lipid, and UA. Abbreviations: ABI: ankle-brachial index; PWV: brachial-ankle pulse wave velocity; AHI: apnea-hypopnea index.

## 4. Discussion

As the main type of diabetes, T2DM has a high morbidity and mortality rate, which brings a certain economic burden to the society and family. OSAHS is a disease characterized by recurrent upper airway stenosis or obstruction during sleep. Complete closure of the upper airway leads to obstructive sleep apnea, and incomplete closure leads to hypopnea. The prevalence of OSAHS in patients with T2DM was significantly higher than that in the general population, and the prevalence of OSAHS in hospitalized T2DM patients was as high as 60% [8]. Patients with coexistence of the two diseases have a significantly higher risk of stroke and cardiovascular disease [9].

Studies have shown that the risk of macrovascular complications in patients with OSAHS and T2DM is significantly increased [10], and the related pathophysiological mechanisms are multifaceted. Intermittent hypoxia and insulin resistance in OSAHS patients can promote the increase of inflammatory markers, including nitric oxide (NO), endothelin-1(ET-1), interleukin-6 (IL-6), and C-reactive protein (CRP). These inflammatory factors affect the vascular endothelial function and participate in inflammatory vascular remodeling and atherosclerosis formation and development. At the same time, studies have found that there are oxidative stress reactions leading to tissue ischemia and hypoxia in patients with OSAHS, including reactive oxygen species (ROS), vascular endothelial growth factor (VEGF), advanced glycation end products (AGEs), and plasminogen activator inhibitor-1 (PAI-1), which leads to the occurrence and development of diabetic vascular disease [11, 12]. Excessive ROS can inhibit insulin-induced energy uptake in fat and muscle tissues, damage islet beta cells, inhibit insulin secretion, and aggravate insulin resistance. Studies have shown that there is upregulated oxidative stress in OSAHS patients, which may have adverse effects on cardiovascular diseases [13]. ET-1 is a vasoconstrictor and can induce inflammatory vascular remodeling, which may be associated with increased cardiovascular risk in patients with OSAHS [14]. VEGF is a hypoxia sensitive glycoprotein. In severe hypoxic patients with OSAHS, the plasma level of VEGF is increased. VEGF stimulates angiogenesis by promoting the proliferation, migration, and proteolysis of vascular endothelial cells [15]. Relevant studies have proved that the level of AGEs in patients with T2DM is increased [16]. The AGE pathway is the main pathophysiological mechanism of diabetic vascular disease development. The accumulation of AGEs in OSAHS may lead to the decrease of endothelial progenitor cells and endothelial repair ability over time, which may lead to the onset of cardiovascular disease.

Several studies have shown the effects of OSA on diabetic macrovascular complications. A longitudinal study in 132 T2DM patients, followed for 4.9 years, revealed that sleep-disordered breathing was a predictor of incident coronary artery disease with a hazard ratio of 1.9 (95% CI: 1.1, 3.3), as well as an increased risk of heart failure [17]. In the Sleep AHEAD study which included 305 T2DM patients, AHI was associated with a 2.57-fold increase in risk of having a history of stroke [18]. A study of 131 patients with T2DM has shown that patients with moderate-to-severe OSAHS and hypertension were three times more likely to have diabetes-related complications compared to those with no or mild OSAHS [19]. In our study, we focused on LEAD as the diabetic macrovascular complications. The results showed that, after adjusting for age, diabetic duration, blood glucose, blood lipid, and other factors, OSAHS was still an independent risk factor of LEAD in patients with T2DM. Also, we further analyzed the influence of different degrees of OSAHS on the occurrence of LEAD in patients. The results showed that mild OSAHS was a predictor of LEAD with a OR of 6.83 (95% CI: 2.28, 20.46) moderate OSAHS with a OR of 27.00 (95% CI: 11.56, 63.08), and severe OSAHS with a OR of 28.07(95% CI: 11.08, 71.12). It was found that with the increasing severity of OSAHS, the risk of LEAD in patients with T2DM was increasing.

Many studies have shown the closely significant relationship between OSAHS and atherosclerosis disease [20, 21]. There are also some studies focused on the association between PWV and AHI [22–24], but the conclusion is inconsistent. A recent study found that in obese patients, AHI was an independent predictor for higher PWV (*r* = 0.352, *p* = 0.038) [22]. Another study also found that the normal-weight sleep breath disorder group had higher PWV than the control group (*p* = 0.03) [23], but a meta-analysis showed that elevated arterial stiffness in patients with OSA is driven by conventional cardiovascular risk factors rather than apnea parameters [24]. However, there are few reports on whether OSAHS affects the early arterial structure and function of patients with T2DM. Clinically, ABI and PWV can reflect the early changes of the arterial structure and function in patients with T2DM. This study found that in T2DM patients with OSAHS, the ABI was significantly lower than that in the control group, and the PWV was significantly increased. At the same time, after adjusting for age, gender, BMI, diabetic duration, blood glucose, blood pressure, blood lipids, and other factors, AHI was still an independent risk factor of increased ABI and decreased PWV. This suggests that the changes of the peripheral arterial structure and function may occur earlier in T2DM patients with OSAHS, and AHI is an independent risk factor of early changes of peripheral arterial disease.

There are some limitations in this study. First of all, the subjects are all hospitalized patients, and the average age of patients in our study is too old to represent all T2DM patients, especially not represent the newly diagnosed T2DM patients. Secondly, the sample size needs to be further expanded. Thirdly, it is only a cross-sectional study of inpatient data, without outpatient data, and lack of longitudinal follow-up data of patients.

## 5. Conclusion

OSAHS is an independent risk factor of atherosclerosis in patients with T2DM, and with the increased severity of OSAHS, the risk of LEAD gradually increases. At the same time, T2DM patients with OSAHS may have early changes of the arterial function, which provides a new clinical basis for the early diagnosis and prevention of LEAD in T2DM patients with OSAHS.

## Figures and Tables

**Table 1:** Comparison of general characteristics, biochemical indices, PSG indices, and atherosclerosis indices among the four groups.

Index	Control group (*n* = 208)	Mild group (*n* = 18)	Moderate group (*n* = 38)	Severe group (*n* = 48)	*F* (*X*^2^)	*p*
Age(y)	47.80 ± 13.61	55.78 ± 9.43^a^	54.32 ± 12.57^a^	52.33 ± 12.86^a^	4.91	<0.05
Gender (male%)	130 (62.5%)	12 (66.67%)	30 (78.95%)	36 (75%)	2.85	0.42
BMI (kg/m^2^)	26.16 ± 3.71	27.20 ± 3.52^a^	27.90 ± 4.97^a^	30.15 ± 4.05^a,b,c^	13.80	<0.05
Diabetic duration (y)	6.51 ± 7.10	8.78 ± 6.59	9.33 ± 7.68	7.78 ± 6.94	2.32	0.19
AHI	2.65 ± 0.78	9.44 ± 2.77^a^	23.26 ± 4.15^a,b^	49.12 ± 13.49^a,b,c^	72.45	<0.05
Minimum SaO_2_ (%)	97.32 ± 5.64	87.76 ± 2.12^a^	81.34 ± 3.47^a,b^	68.85 ± 2.66^a,b,c^	7.86	<0.05
SBP (mmHg)	130.58 ± 16.10	140.00 ± 13.12^a^	140.63 ± 12.05^a^	140.83 ± 11.72^a^	10.49	<0.05
DBP (mmHg)	79.39 ± 11.41	81.00 ± 10.00	79.84 ± 10.96	82.79 ± 11.09	1.24	0.30
FBG (mmol/l)	9.18 ± 3.56	8.11 ± 2.79	8.60 ± 3.07	9.43 ± 3.78	0.41	0.74
PBG (mmol/l)	13.20 ± 5.67	14.15 ± 5.34	14.19 ± 6.94	13.08 ± 3.62	0.44	0.72
HbA1c (%)	8.54 ± 1.96	8.11 ± 1.62	8.38 ± 1.61	8.31 ± 1.79	0.43	0.73
TC (mmol/l)	4.53 ± 1.14	4.48 ± 0.95	4.14 ± 0.93	4.62 ± 1.47	1.41	0.24
TG (mmol/l)	2.52 ± 1.83	2.03 ± 1.13	1.82 ± 0.74	2.52 ± 1.83	2.13	0.10
LDL-C (mmol/l)	2.65 ± 0.87	2.74 ± 0.76	2.32 ± 0.79	2.80 ± 1.23	2.06	0.11
HDL-C (mmol/l)	0.96 ± 0.24	0.91 ± 0.28	0.91 ± 0.26	0.94 ± 0.16	0.47	0.70
UA (umol/l)	346.52 ± 89.89	359.22 ± 91.36	397.11 ± 79.48^a,b^	381.14 ± 77.22^a^	4.79	<0.05
eGFR (ml/min/1.73m^2^)	102.49 ± 18.04	96.26 ± 16.86	95.16 ± 14.41	98.64 ± 17.14	2.55	0.06
UACR (mg/g)	21.57 ± 39.81	37.97 ± 41.95	32.65 ± 42.08	31.94 ± 59.40	1.70	0.17
ABI	1.15 ± 0.09	1.14 ± 0.06^a^	1.14 ± 0.09^a^	1.05 ± 0.15^a,b,c^	12.34	<0.05
PWV (cm/s)	1435.05 ± 289.29	1487.73 ± 283.90^a^	1720.08 ± 281.15^a^	1801.89 ± 498.59^a,b,c^	17.62	<0.05
LEAD (%)	18 (8.65%)	8 (44.44%)^a^	18 (47.36%)^a^	34 (70.83%)^a,b,c^	61.75	<0.05

^a^ *p* < 0.05 compared with the control group, ^b^*p* < 0.05 compared with the mild group, ^c^*p* < 0.05 compared with the moderate group. Abbreviations: SBP: systolic blood pressure; DBP: diastolic blood pressure; BMI: body mass index; FBG: fasting blood glucose; PBG: postprandial blood glucose; HbA1c: glycosylated hemoglobin; UA: uric acid; TC: total cholesterol, TG: triglyceride; LDL-C: low-density lipoprotein cholesterol; HDL-C: high-density lipoprotein cholesterol; ABI: ankle-brachial index; PWV: brachial-ankle pulse wave velocity; AHI: apnea-hypopnea index; LEAD: lower extremity arterial disease.

**Table 2:** Linear correlation analysis between AHI and general characteristics, biochemical indices, ABI, and PWV.

	AHI			AHI	
Index	*R*	*p*	Index	*R*	*p*
Age (y)	0.02	<0.05	TC (mmol/l)	0.30	<0.05
Diabetic duration (y)	-0.18	0.06	TG (mmol/l)	0.28	<0.05
BMI (kg/m^2^)	0.32	<0.05	LDL-C (mmol/l)	0.34	<0.05
SBP (mmHg)	0.11	0.27	HDL-C (mmol/l)	0.03	0.74
DBP (mmHg)	0.14	0.16	eGFR (ml/min/1.73m^2^)	0.05	0.64
FBG (mmol/l)	0.11	0.30	UACR (mg/g)	0.10	0.32
PBG (mmol/l)	0.13	0.23	UA (umol/l)	0.17	0.10
HbA1c (%)	0.04	0.73	ABI	-0.37	<0.05
			PWV (cm/s)	0.36	<0.05

Abbreviations: SBP: systolic blood pressure; DBP: diastolic blood pressure; BMI: body mass index; FBG: fasting blood glucose; PBG: postprandial blood glucose; HbA1c: glycosylated hemoglobin; UA: uric acid; TC: total cholesterol, TG: triglyceride; LDL-C: low-density lipoprotein cholesterol; HDL-C: high-density lipoprotein cholesterol; AHI: apnea-hypopnea index; ABI: ankle-brachial index; PWV: brachial-ankle pulse wave velocity.

**Table 3:** Multiple stepwise linear regression results between AHI level with ABI and PWV.

	ABI			PWV		
Index	*β*st	*t*	*p*	*β*st	*t*	*p*
Model 1						
AHI	0.01	5.34	*p* < 0.05	26.26	6.46	*p* < 0.05
Model 2						
AHI	0.01	5.15	*p* < 0.05	27.24	6.16	*p* < 0.05
Model 3						
AHI	0.01	5.19	*p* < 0.05	26.92	5.68	*p* < 0.05

Model 2 is adjusted for age, gender, and BMI; model 3 is adjusted for age, gender, diabetic duration, BMI, blood glucose, blood pressure, blood lipid, and UA. Abbreviations: ABI: ankle-brachial index; PWV: brachial-ankle pulse wave velocity; AHI: apnea-hypopnea index.

**Table 4:** Multivariate logistic regression results of OSAHS and LEAD in patients with T2DM.

	LEAD		
Index	OR	95% CI	*p*
Model 1			
Control group	1	1	
Mild group	8.44	2.96, 24.08	*p* < 0.05
Moderate group	25.63	11.66, 56.37	*p* < 0.05
Severe group	29.56	12.40, 70.47	*p* < 0.05
Model 2			
Control group	1	1	
Mild group	6.83	2.28, 20.46	*p* < 0.05
Moderate group	27.00	11.56, 63.08	*p* < 0.05
Severe group	28.07	11.08, 71.12	*p* < 0.05

## Data Availability

The data used to support the findings of this study are available from the corresponding author and first author upon request.
